# Prevalence and Related Factors of Insomnia Among Chinese Medical Staff in the Middle and Late Stage of COVID-19

**DOI:** 10.3389/fpsyt.2020.602315

**Published:** 2020-12-07

**Authors:** Dianying Liu, Shaohua Liu, Lin Zhu, Dongbin Li, Donghua Huang, Hongdong Deng, Huiyun Guo, Dan Huang, Yuanping Liao, Zhongzhen Mao, Qiumei Miao, Wanglin Liu, Meihong Xiu, Xiangyang Zhang

**Affiliations:** ^1^Department of Psychiatry, The Third People's Hospital of Ganzhou City, Ganzhou, China; ^2^School of Education Science, Gannan Normal University, Ganzhou, China; ^3^Beijing HuiLongGuan Hospital, Peking University HuiLongGuan Clinical Medical School, Beijing, China; ^4^Chinese Academy of Sciences Key Laboratory of Mental Health, Institute of Psychology, Chinese Academy of Sciences, Beijing, China

**Keywords:** insomnia, COVID-19, prevalence, resilience, medical health staff

## Abstract

**Background:** The outbreak of novel coronavirus disease (COVID-19) has brought serious psychological pressure to people, especially medical health staff. At present, there are few studies on insomnia and related factors of medical health staff in the middle and late stage of the epidemic of COVID-19. Therefore, the purpose of this study was to investigate the prevalence of insomnia and its related risk factors among medical workers in China in the middle and later stage of COVID-19 epidemic, as well as the relationship between insomnia and psychological resilience.

**Methods:** From February 14 to March 29, 2020, a cross-sectional survey was conducted among 606 medical staff in China through Ranxing Technology's “SurveyStar” network platform. All subjects were assessed with the Insomnia Severity Index (ISI) and simplified Chinese version of Connor-Davidson Resilience scale (CD-RISC-10).

**Results:** In the middle and later stages of the COVID-19 outbreak, the incidence of insomnia among medical staff was 32.0%. Compared with non-insomnia group, the insomnia group had younger age, lower education level, longer daily working hours and less psychological resilience. In addition, the prevalence of insomnia was higher in medical staff with a history of somatic diseases. The severity of insomnia of Chinese medical staff was associated with age, education level, daily working hours, psychological resilience and somatic diseases.

**Conclusions:** Our study shows that nearly 1/3 of Chinese medical workers suffer from insomnia nearly a month after the COVID-19 outbreak. Compared with the general population, medical staff who are working with COVID are more prone to insomnia. Risk factors for insomnia include younger age, lower education level, longer working hours per day, and physical illness. The tenacious dimension of psychological resilience is a protective factor for insomnia.

## Introduction

Insomnia is a major mental problem for medical staff, especially during disease outbreaks. Previous studies have shown that during the SARS epidemic, the percentage of insomnia among Chinese health care workers ranged from 37.1% (1) to 52.3% (2–4), which was 5–7 times (6–12.2%) higher than the public population (2, 4, 5). In December 2019, the novel coronavirus disease (COVID-19) first appeared in Wuhan, Hubei, China (6), and then spread to other cities and even abroad (7). Because of its high infectivity and fatality rate, it brings tremendous psychological pressure to medical staff, which leads to an increase in the incidence of insomnia and seriously affects the mental health of medical staff (8, 9). In addition, insomnia is a major risk factor that increases depression, anxiety and suicide (10, 11).

A variety of common factors, such as age (4, 8, 9, 12, 13), women (4, 9, 12, 13), low education level (4, 9, 13), low income level (4), isolation environment (9), and marital status (4, 12, 13) has been shown to be associated with personal insomnia. Moreover, another risk factor for insomnia among medical staff includes job position, such as nurse (9). Other studies have shown that long-term working hours and steady-state overload are important factors for increased insomnia among medical health workers (1, 8, 9, 14–16). Taken together, insomnia is caused by different risk factors, and insomnia is still a major health problem for medical workers. Most previous studies have focused on the relationship between insomnia and the sociodemographic variables, and only a few studies have investigated the relationship between insomnia and psychological resilience (17–21). Psychological resilience is the ability to adapt to stress and adversity. Li et al. (18) and Cheng et al. (21) found that people with low resilience directly lead to poor sleep quality. Similarly, Brand et al. (22) reported that compared with the high resilience group, the low resilience group has lower sleep efficiency, more awakenings, and lighter sleep. A recent study also found a significant positive correlation between the resilience and the quality of sleep in pregnant woman (17). However, most of those studies were conducted among non-medical staff. Therefore, whether psychological resilience is a protective factor for sleep quality of medical staff is still unknown and deserves further study.

To our best knowledge, no study has reported the relationship between psychological resilience and insomnia of medical staff among Chinese Han population. Therefore, this study aimed to determine the prevalence and related factors of insomnia in Chinese medical staff through a cross-sectional design, including demographic data, daily working hours, somatic diseases, and psychological resilience.

## Methods

### Subjects and Settings

A cross-sectional study was conducted from February 14 to March 29 2020. All questionnaires were distributed in the form of posters through the “SurveyStar” network platform (Ranxing Technology), which was forwarded through Wechat and other channels. The recruited medical staff logged in by scanning the QR code and filled in the questionnaire. Finally, a total of 606 valid data were collected.

The research protocol was approved by the Ethics Committee of the Institute of Psychology, Chinese Academy of Sciences.

### Measurements

A structured self-assessment questionnaire was conducted in this survey. Demographic data of all subjects were collected, including sex, age, height, weight, marital status, education level, occupation (doctors, nurses, or medical technicians), daily working hours, history of SARS epidemic in 2003, annual family income, history of somatic diseases, COVID-19 infection of relatives and friends, economic losses caused by COVID-19. The Chinese version of the Insomnia Severity Index (ISI) (23) was used to assess the severity of insomnia of all subjects. ISI is a self-report scale composed of 7 items. Each item is evaluated on a five-point Likert scale (from 0 = not at all to 4 = extremely), and the usual recall interval is the “last 2 weeks.” The total score ranges from 0 to 28, and the higher the score, the greater the severity of insomnia. According to a previous study (24) a total score of 0–7 indicated no insomnia, 8–14 indicated sub-threshold insomnia, 15–21 indicated moderate insomnia, and 22–28 indicated severe insomnia. In this survey, a total score of ISI ≥ 8 indicated insomnia (9, 25).

Psychological resilience was measured by the simplified Chinese version of the 10- item Connor-Davidson Resilience Scale (CD-RISC-10). CD-RISC-10 is an important tool for assessing an individual's ability to cope with stress and rebound from difficult events or experiences (26), and has been widely used all over the world and in different populations (27). CD-RISC-10 is a five-point Likert (from 0 = never, 1 = rarely, 2= sometimes, 3 = often, and 4 = always) and a self-rating scale. The usual recall interval is “the past month.” The total score ranges from 0 to 40, and a high total score indicates greater resilience (28). This scale includes two factors. One is strength, which mainly indicates the ability to deal with difficult problems, or the ability to achieve goals, and physical and mental resilience, which is composed of five items. The other is hardiness, which mainly refers to the ability to adapt to change in the face of setbacks or adversity, consisting of five items (29). The total score of these two factors ranges from 0 to 20 points.

### Statistical Analyses

The demographic and mental health variables of insomnia and non-insomnia groups were compared using independent samples *t*-tests for continuous variables and chi-square for categorical variables. The prevalence of insomnia was described by percentage and analyzed by chi-square test. Binary logistic regression analysis was performed to assess which factors were significantly associated with insomnia. The correlation between insomnia and demographic and mental health variables was conducted by Pearson or Spearman correlation coefficients. Bonferroni correction was performed for each test to adjust for multiple tests. Then stepwise multiple regression analysis was applied to explore the significant predictive variables related to insomnia. IBM SPSS 22.0 was performed for all statistical analysis. All *p*-values were two tailed and the significance level was < 0.05.

## Results

### Demographic and Mental Health Characteristics

The demographic and mental health characteristics of all subjects are shown in Table 1. There were 114 males (18.8%) and 492 females (81.2%). The average age of all subjects was 35.77 years old, ranging from 20 to 65 years old. The average body mass index (BMI) was 22.76 kg/m^2^, ranging from 12.36 to 47.27 kg/m^2^. 152 subjects (25.08%) were single and 454 (74.91%) were married. There were 80 (13.2%) subjects with a college degree or below, 380 (62.7%) with a bachelor degree, 92 (15.2%) with a master degree, and 54 (8.9%) with a doctoral degree. Among the jobs, there were 205 doctors (33.8%), 334 nurses (55.1%), and 67 medical technicians (11.1%). Eighty-four subjects (46.9%) worked <8 h a day, 268 subjects (44.2%) worked for 10 h or less, and 54 subjected (8.9%) worked for more than 10 h a day. In addition, the annual household income of 106 subjects (17.5%) was 30,000–80,000 yuan, 402 (66.3%) were 80,000–300,000 yuan, and 98 (16.2%) were 300,000–1,000,000 yuan. In 2003, 262 subjects (43.2%) experienced the SARS epidemic. 137 subjects (22.6%) had a history of somatic disease. The average CD-RISC-10 score of all subjects was 27.05. The average strength factor score was 13.66, and the average hardiness factor score was 13.39.

**Table 1:** Demographic and mental health characteristics of medical staff with or without insomnia.

	**Total**	**Non-insomnia group**	**Insomnia group**	***t*/χ^**2**^**	***p*-Value**
	**606**	**(*n* = 412, 68.0%)**	**(*n* = 194, 32.0%)**		
**Sex**				0.11	0.739
Male, *n* (%)	114 (18.8)	79/114 (69.30)	35/114 (30.70)		
Female, *n* (%)	492 (81.2)	333/492 (67.68)	159/492 (32.32)		
Age, years, M (SD)	35.77 (8.13)	38.45 (8.08)	34.33 (8.07)	3.01	0.003
BMI, M (SD)	22.76 (3.81)	22.56 (3.44)	23.18 (4.47)	−1.85	0.065
Marital status				5.19	0.023
Single, *n* (%)	152 (25.08)	92/152 (60.5)	60/152 (39.5)		
Married, *n* (%)	454 (74.91)	320/454 (70.5)	134/454 (29.5)		
Education				9.59	0.022
College degree and below, *n* (%)	80 (13.2)	45/80 (56.2)	35/80 (43.8)		
Bachelor degree, *n* (%)	380 (62.7)	256/380 (67.4)	124/380 (32.6)		
Master degree, *n* (%)	92 (15.2)	69/92 (75.0)	23/92 (25.0)		
Doctoral degree, *n* (%)	54 (8.9)	42/54 (77.8)	12/54 (22.2)		
**Job position**				12.56	0.002
Doctors	205 (33.8)	153 (74.6)	52 (25.4)		
Nurses	334 (55.1)	207 (62.0)	127 (38.0)		
Medical technicians	67 (11.1)	52 (77.6)	15 (22.4)		
Working hours/day (h)				12.09	0.002
≦8	284 (46.9)	208 (73.2)	76 (26.8)		
8–10	268 (44.2)	177 (66.0)	91 (34.0)		
>10	54 (8.9)	27 (50.0)	27 (50.0)		
**History of SARS epidemic in 2003**				1.92	0.166
No	344 (56.8)	226 (65.7)	118 (34.3)		
Yes	262 (43.2)	186 (71.0)	76 (29.0)		
Annual household incomes (Yuan)				7.03	0.03
30,000–80,000	106 (17.5)	61 (57.5)	45 (42.5)		
80,000–300,000	402 (66.3)	279 (69.4)	123 (30.6)		
300,000–≥1,000,000	98 (16.2)	72 (73.5)	26 (26.5)		
History somatic disease				**8.67**	**0.003**
No	469 (77.4)	333 (71.0)	136 (29.0)		
yes	137 (22.6)	79 (57.7)	58 (42.3)		
**CD-RISC-10**					
Total score, M (SD)	27.05 (8.71)	28.67 (8.65)	23.60 (7.80)	6.94	<0.001
Strength factor, M (SD)	13.66 (4.51)	14.45 (4.47)	11.97 (4.13)	6.51	<0.001
Hardiness factor, M (SD)	13.39 (4.35)	14.22 (4.33)	11.63 (3.84)	7.12	<0.001

### Prevalence of Insomnia and Comparison of Demographic and Mental Health Variables Between Insomnia and Non-insomnia Participants

As shown in Table 1, the prevalence of insomnia among medical staff was 32.0% (194/606). The average age of the insomnia group was significantly younger than that of the non-insomnia group (*p* = 0.003). The prevalence of insomnia in single subjects was significantly higher than that in married subjects (*p* = 0.023). The prevalence of insomnia in college degree and below was significantly higher 43.8% (35/80) than that bachelor degree 32.6% (124/380), master degree 25.0% (23/92) and doctoral degree 22.2% (12/54) (*p* = 0.022, Bonferroni corrected *p* < 0.05). The prevalence of insomnia among nurses was significantly higher than that in doctors and medical technicians (*p* = 0.002, Bonferroni corrected *p* < 0.01). The incidence of insomnia in the group working more than 10 h a day was significantly higher than that in the 8–10 h group and the 8 h group (*p* = 0.002, Bonferroni corrected *p* < 0.01). The rate of insomnia in medical staff with somatic disease was significantly higher than that in non-somatic disease group (*p* = 0.003).

The total score, strength factor and hardiness factor scores of CD-RISC-10 in insomnia group were significantly lower than those in non-insomnia group (all *p* < 0.001). There was no significant difference in BMI, history of SARS epidemic in 2003 (all *p* > 0.05). The lowest annual household incomes group had significantly highest rate of insomnia (*p* = 0.03) (Table 1). In addition, after controlling for gender as a covariate, these differences remained significant (all *p* < 0.05).

### Correlation of Insomnia and Demographic and Mental Health Measures

The average ISI total score in all medical staff was 6.27 ± 6.13. Pearson correlation analysis showed that ISI was correlated with age (*r* = −0.134, *p* < 0.001), marital status (*r* = −0.086, *p* < 0.05), education level (*r* = −0.143, *p* < 0.001), daily working hours (*r* = 0.1, *p* < 0.01), physical illness (*r* = 0.095, *p* < 0.05), strength factor (*r* = −0.304, *p* < 0.001), and hardiness factor (*r* = −0.327, *p* < 0.001). Further, except for marital status, all these associates remained significant (*p* < 0.05) after the Bonferroni correction. Table 2 shows the association between ISI and demographic data or mental health variables.

**Table 2:** Association between ISI and demographic data and mental health variables.

**Variables**	**ISI**	**Sex**	**Age**	**Marital**	**Education**	**Job position**	**Working duration**	**Annual incomes**	**Somatic disease**	**Resilience**	**Strength**	**Hardiness**
ISI	1											
Sex	0.052	1										
Age	−0.134^***^	−0.172^***^	1									
Marital	−0.086^*^	−0.074	0.480^***^	1								
Education degree	−0.143^***^	−0.193^**^	0.275^***^	0.158^***^	1							
Job position	0.069	0.322^**^	−0.220^***^	−0.119^**^	−0.381^***^	1						
Working duration	0.100^**^	−0.081*	0.115^**^	0.031	0.131^**^	−0.185^***^	1					
Annual incomes	−0.070	−0.011	0.295^***^	0.249^***^	0.376^***^	−0.117^***^	0.115^**^	1				
Somatic disease	0.095^*^	−0.002	0.217^***^	0.103^**^	0.035	−0.049	0.092^*^	0.094^*^	1			
Resilience	−0.321^***^	−0.064	0.218^***^	0.045	0.176^***^	−0.053	0.011	0.176^***^	0.022	1		
Strength	−0.304^***^	−0.050	0.210^***^	0.037	0.167^***^	−0.049	0.006	0.178^***^	0.020	0.983^***^	1	
Hardiness	−0.327^***^	−0.076	0.217^***^	0.051	0.179^***^	−0.055	0.16	0.168^***^	0.024	0.982^***^	0.932^***^	1

* Indicates that there was a significant corelation.

* P < 0.05;

** p < 0.01;

*** *p < 0.001*.

### Factors Associated With Insomnia

Multiple stepwise regression was performed to identify demographic and mental health variables that were associated with ISI. There were five variables that statistically predicted ISI [*F*_(5, 600)_ = 20.05*, p* < 0.001, *r*^2^ = 0.136], including hardiness factor, daily working hours, education level, physical illness, and age. The coefficients of these variables are shown in Table 3.

**Table 3:** Predictors generated by multivariate logistic regression with ISI total score as dependent variables.

	**Coefficients**		**Standardized coefficients**	***T***	***p*-Value**	**95.0% confidence interval for B**	
	***B***	**Std. Error**	**Beta**			**Lower bound**	**Upper bound**
(Constant)	13.479	1.290		10.450	0.000	10.946	16.012
Hardiness factor	−0.420	0.055	−0.298	−7.636	0.000	−0.529	−0.312
Working hours/day	1.097	0.365	0.115	3.004	0.003	0.380	1.815
Education	−0.679	0.315	−0.086	−2.154	0.032	−1.297	−0.060
Somatic disease	1.652	0.569	0.113	2.904	0.004	0.535	2.769
Age	−0.063	0.031	−0.083	−2.022	0.044	−0.123	−0.002

## Discussion

To our best knowledge, this is the first study to investigate the percentage of insomnia and its related risk factors among medical staff under the long-term influence of COVID-19 pandemic in China, as well as the relationship between insomnia and psychological resilience. The main findings of this survey included: (1) the percentage of insomnia in medical staff was 32.0%; (2) medical staff working with COVID were more prone to insomnia than the general population; (3) the risk factors of insomnia in medical staff were younger age, lower education level, longer working hours per day, and physical illness; (4) hardiness factor of psychological resilience was the protective factor for insomnia of medical staff.

Our cross-sectional study indicated that during the COVID-19 epidemic in China, the percentage of medical staff who suffered from insomnia was 32.0%, which was lower than the previous studies of 34.0% (30), 36.1% (9), 38.4% (31), but was relatively higher than the 30.5% prevalence of non-medical personnel under the COVID-19 epidemic (31). The difference in the incidence of insomnia was most likely to be related to the following reasons. First, contrary to previous studies, the duration of our investigation was longer, from February 14 to March 29, 2020. At the beginning of the COVID-19 outbreak, medical staff lacked awareness of the disease and lacked protective equipment, which increased their anxiety, fear, and insomnia. With the spread of the COVID-19, medical staff has had a better understanding of the disease, treatment for the disease has been improved, and anxiety, fear and insomnia have been alleviated. Second, more medical staff have been sent to Wuhan, reducing the pressure on medical staff. Third, the government and various units have taken a series of timely and effective psychosocial interventions and support measures (6). Taken together, these studies have shown that with the progression of the COVID-19 epidemic, the prevalence of insomnia gradually decreases, but is still higher in medical staff than that of the Chinese public during the COVID-19 epidemic.

Furthermore, our study found that the ISI total score was negatively correlated with age in Chinese medical staff, which is consistent with previous studies showing that medical staff in the insomnia group were between 18 and 25 years old (9). As pointed out by Huang and Zhao (8), during COVID-19 outbreak, younger participants are more likely to suffer from anxiety and depressive than older people. The possible reason is that young medical have relatively lack of clinical experience in the face of inadequate working environment, including long waiting lists of patients, heavy workload, insufficient resources, and daily working overload, which can easily lead to anxiety, depression, insomnia and other problems. But gender was not related to the insomnia during COVID-19 outbreak, which was consistent with previous studies (8, 9).

In addition, we found that marital status was associated with insomnia, which is in line with a previous study (9) reporting that the insomnia rate of single medical staff was higher than that of married subjects (38.1 vs. 34.43%). However, other studies showed that married participants have a higher rate of insomnia than single participants (12, 13, 32). The possible reason for these inconsistent results is that the participants come from different places. The subjects recruited in this study and Zhang et al. (9) study were hospital staff from all over the country, including front-line medical staff. But the subjects of Li et al. (32) all came from Ningbo city. In another study, the subjects were from the general population (13). Therefore, it is necessary to further investigate the differences in the incidence of insomnia among medical staff with different marital status. In addition, we found that medical staff with lower education level were at a higher risk of insomnia, which was consistent with several previous studies (9, 13) showing that low education level was associated with a high risk of insomnia in the general population and medical staff in China. The main reason is that it is more difficult for medical staff with low education level to grasp the information related to disease outbreaks, and their working ability is relatively poor. During the COVID-19 outbreak, they had a stronger fear of the disease, which may affect their sleep quality (9). It is worth noting that our study further indicated that medical staff who had longer working hours per day reported more serious symptoms of insomnia. This was in line with previous studies showing that medical staff who worked longer than usual reported more severe insomnia (30, 33). A potential explanation is that during the COVID-19 epidemic, both frontline and non-frontline medical staff spent a lot of time participating in the antiepidemic work, irregular shifts, excessive workload and longer working hours made them more stressed than usual (16, 32). These stresses constituted a source of unbalanced load, leading to burnout syndrome (BS) (34) and may significantly impair sleep (15, 31). As expected, insomnia was significantly associated with somatic diseases in medical staff, and with the increase in comorbid somatic diseases in the entire sample, the prevalence of insomnia became higher. Consistent with other studies, patients with chronic physical diseases have an increased risk of insomnia (31, 35). Whether medical staff or non-medical staff, organic diseases are independent risk factors for insomnia (9). One possible explanation is that physical complaints, such as headache and cardiovascular disease are more likely to enhance autonomic hyperarousal. Second, during the COVID-19 epidemic, medical staff with physical diseases may worry about infection, resulting in more serious symptoms of anxiety and insomnia. Moreover, negative thoughts about threatening symptoms may lead to persistent insomnia (36).

Another important finding of this study was that all the dimensions and total score of psychological resilience were significantly negatively correlated with the ISI total score. Our study also showed that non-insomnia medical staff had better psychological resilience and stronger strength and hardiness. This was in accordance with other studies showing that participants with low resilience directly lead to poor sleep quality (17, 20), such as lower sleep efficiency, more awakening times, and lighter sleep than those with higher resilience (22).

However, in this study, multiple stepwise regression found that only the hardiness subscale of the psychological resilience was the significant protective factor for insomnia in medical workers. Hardiness reflects a person's ability to rebound from adversity, emotional control, decision-making, and problem-solving. Previous study reported that hardiness was a protective factor for negative health outcomes among the five factors of resilience (hardiness, optimism, persistence, support, and spirituality) (37). These findings indicate that psychological resilience is a protective factor for insomnia in medical staff.

Our research had several limitations. First, this survey adopted a cross-sectional design, based on the WeChat program and self-administered questionnaire. Second, the duration of the survey is comparatively short, which cannot effectively verify whether there is dynamic balance overload as COVID-19 progresses. Third, only ISI was used to assess the severity of insomnia. It is possible that many other sleep problems had not been assessed. Fourth, we did not compare the difference in insomnia between frontline and non-frontline medical workers. It is not obvious whether the data on insomnia in medical staff working with COVID is different from the insomnia in medical workers dealing with routine diseases. Fifth, the pre-epidemic status was not collected, which may lead to biased results. In addition, the most important limitation is the source of recruitment. This sample could be not representative because the older medical staff could not use social networks. This is evident because of low mean age of participants (35 years).

In conclusion, this study revealed that during the COVID-19 outbreak, the prevalence of insomnia among medical staff was higher than that of the Chinese public. The related risk factors included younger age, lower education level, physical disease, and longer working hours per day. In addition, the hardiness of psychological resilience was a protective factor for insomnia of medical staff. Therefore, when carrying on the psychological intervention to the medical staff, we need to consider different social and psychological factors. The most important intervention is to improve the psychological adaptability of medical staff.

## Data Availability Statement

The original contributions presented in the study are included in the article/supplementary material, further inquiries can be directed to the corresponding author/s.

## Ethics Statement

The studies involving human participants were reviewed and approved by the Ethics Committee of the Institute of Psychology, Chinese Academy of Sciences. The patients/participants provided their written informed consent to participate in this study.

## Author Contributions

XZ: conceptualization, methodology, writing—reviewing, editing, and supervision. DLiu: formal analysis, writing—original draft, and funding acquisition. SL, LZ, DLi, DoH, HD, HG, DaH, YL, and ZM: investigation, resources, and data curation. WL, QM, and MX: conceptualization, writing—review, and editing. All authors contributed to the article and approved the submitted version.

## Conflict of Interest

The authors declare that the research was conducted in the absence of any commercial or financial relationships that could be construed as a potential conflict of interest.
